# Occurrence of plasmid-mediated quinolone resistance genes in *Pseudomonas aeruginosa* strains isolated from clinical specimens in southwest Iran: a multicentral study

**DOI:** 10.1038/s41598-022-06128-4

**Published:** 2022-02-10

**Authors:** Morteza Saki, Ahmad Farajzadeh Sheikh, Sakineh Seyed-Mohammadi, Aram Asareh Zadegan Dezfuli, Mojtaba Shahin, Maryam Tabasi, Hojat Veisi, Raziyeh Keshavarzi, Parisa Khani

**Affiliations:** 1 Infectious and Tropical Diseases Research Center, Health Research Institute, Ahvaz Jundishapur University of Medical Sciences, Ahvaz, Iran; 2 Department of Microbiology, Faculty of Medicine, Ahvaz Jundishapur University of Medical Sciences, Ahvaz, Iran; 3 Student Research Committee, Ahvaz Jundishapur University of Medical Sciences, Ahvaz, Iran; 4 Department of Medical Laboratory Sciences, Faculty of Medical Sciences, Arak Branch, Islamic Azad University, Arak, Iran; 5 Department of Virology, Faculty of Medicine, Ahvaz Jundishapur University of Medical Sciences, Ahvaz, Iran

**Keywords:** Antimicrobial resistance, Epidemiology

## Abstract

This study aimed to assess the presence of *qnrA*, *qnrB*, *qnrC*, *qnrD*, *qnrS*, *qepA*, and *aac(6′)-Ib-cr* determinants as well as quinolone resistance pattern of clinical isolates of *P. aeruginosa* in Ahvaz, southwest Iran. A total of 185 clinical isolates of *P. aeruginosa* were collected from 5 university-affiliated hospitals in Ahvaz, southwest Iran. The disk diffusion method was applied to assess the quinolone resistance pattern. The presence of *qnrA*, *qnrB*, *qnrC*, *qnrD*, *qnrS*, *qepA*, and *aac(6′)-Ib-cr* genes was investigated by the polymerase chain reaction (PCR) method. Overall, 120 (64.9%) isolates were non-susceptible to quinolones. The most and the less quinolone resistance rates were observed against ciprofloxacin (59.4%) and ofloxacin (45.9%), respectively. The prevalence rates of *qnr* genes were as follows: *qnrA* (25.8%), *qnrB* (29.2%), and *qnrS* (20.8%). The *qnrB* gene was the most common type of *qnr* genes. The *qnr* genes were occurred in 37.5% (n = 45/120) of quinolne-resistant isolates, simultaneously. The *qnrC*, *qnrD*, *qepA*, and *aac(6′)-Ib-cr* genes were not recognized in any isolates. In conclusion, the ofloxacin was the most effective quinolone. This study was the first to shed light on the prevalence of PMQR genes among *P. aeruginosa* isolates in southwest Iran.

## Introduction

*Pseudomonas aeruginosa* as one of the most frequent nosocomial pathogens has become an important leading cause of death in burn, cystic fibrosis (CF), and immunocompromised patients^1–3^. According to the records of the Centers for Disease Control (CDC), approximately 51,000 nosocomial *P. aeruginosa* infections occur annually in the United States^4^. Nearly, 13% of them are caused by multidrug-resistant (MDR) *P. aeruginosa* species that account for about 400 deaths per year^4^. The widespread use of broad-spectrum antibiotics has resulted in the emergence of MDR bacterial strains as a major problem in health care systems^5^. It has been remarked that the MDR *P. aeruginosa* strains are associated with a considerable increase in the length of hospitalization as well as morbidity and mortality^6^. The most effective antibacterial compounds against *P. aeruginosa* include β-lactams, aminoglycosides, and fluoroquinolones^7^.

The use of fluoroquinolone antibiotics has spread widely in the past decade leading to the emergence of resistant bacterial strains^8,9^. The plasmid-mediated quinolone resistance (PMQR) is considered as a common mechanism contributed to resistance among Gram-negative bacilli^10,11^. The PMQR was initially reported in 1998 from the clinical isolates of *Klebsiella pneumoniae*. So far, three PMQR mediated mechanisms were recognized that include *qnr* genes (coding Qnr proteins), the acetyltransferase *aac(6′)-Ib-cr* which is a variant of an enzyme involved in aminoglycoside alteration and resistance, and active efflux pumps such as QepA and OqxAB^10,11^. So far, seven major Qnr families have been identified: QnrA, QnrB, QnrS, QnrC, QnrD, QnrE, and QnrVC^12^. The *qnr* genes counteract with the blockage effects of quinolone antibiotics on the microbial enzymes such as topoisomerase II (DNA gyrase) and topoisomerase IV^10^. Other probable quinolone resistance mechanisms in *P. aeruginosa* include chromosomal mutations in quinolone resistance determining region (QRDR) of topoisomerase (*parC/parE*) and DNA gyrase (*gyrA*/*gyrB*) encoding genes, and mobile efflux pumps such as OqxAB^10–14^. Also, mutations of the regulatory genes that affect the permeability or efflux process are among the contributed quinolone resistance mechanisms^10,11^.

To the best of our knowledge, there are scarce data available on the prevalence of PMQR genes among clinical isolates of *P. aeruginosa* worldwide, especially in Iran. Thus, considering the importance of *P. aeruginosa* and the *qnr* genes, the present study aimed to assess the prevalence of *qnrA*, *qnrB*, *qnrS*, *qnrC*, *qnrD*, *qepA*, and *aac(6′)-Ib-cr* genes as well as the antibiotic resistance pattern of fluoroquinolone-resistant *P. aeruginosa* strains isolated from clinical specimens in Ahvaz city, southwest of Iran.

## Results

### Bacterial isolates

Based on standard bacteriological tests, overall, 185 (22.5%) clinical strains of *P. aeruginosa* were collected from four main hospitals in Ahvaz, southwest of Iran during a two-year period. All isolates were positive for *ecfX* gene. The distribution of *P. aeruginosa* isolates according to hospital, gender, age, wards, and specimens are summarized in Table 1. The cases included 75 (40.5%) females and 110 (59.5%) males. The bacterial isolates were obtained from different clinical specimens. The most isolates were collected from wound, n: 69 (37.3%); followed by urine, n: 40 (21.6%); blood, n: 36 (19.4%); abscess, n: 15 (8.1%); sputum, n: 12 (6.5%); abdominal secretion, n: 8 (4.3%); and cerebrospinal fluid, n: 5 (2.7%). Also, the majority of isolates were collected from Burn, Urology, and Intensive Care Unit (ICU) ward.

**Table 1:** Distribution of the *Pseudomonas aeruginosa* isolates according to the hospital, gender, age, wards, and specimens.

Sources	No. (%) of strains
**Hospital**	
Golestan	65 (45.9)
Taleghani	55 (29.7)
Razi	27 (14.5)
Imam Khomeini	38 (20.5)
**Gender**	
Male	110 (59.5)
Female	75 (40.5)
**Age (year)**	
< 10	45 (24.3)
10–19	29 (15.7)
20–29	19 (10.3)
30–39	18 (9.7)
40–49	32 (17.3)
50–59	15 (8.1)
> 60	27 (14.6)
**Wards**	
Pediatric	15 (8.1)
Outpatient Department	10 (5.4)
Intensive Care Unit	26 (14)
Urology	30 (16.2)
Surgery	19 (10.3)
Neonatal Intensive Care Unit	12 (6.5)
Bone marrow transplantation	4 (2.2)
Cardiovascular	3 (1.6)
Hematology	14 (7.6)
Gastrointestinal	3 (1.6)
Burn	30 (16.2)
Infectious diseases	19 (10.3)
**Specimens**	
Urine	40 (21.6)
Blood	36 (19.4)
Wound	69 (37.3)
Sputum	12 (6.5)
Abdominal secretion	8 (4.3)
Cerebrospinal fluid	5 (2.7)
Abscess	15 (8.1)

### Resistance to quinolone compounds

Overall, 120 (64.9%) isolates were non-susceptible to quinolone compounds used in this study. The antibacterial susceptibility testing revealed that ofloxacin (54.1% susceptible) was the most effective drug compared to the other fluoroquinolone antibiotics. The highest resistance rates of isolates were against ciprofloxacin (59.4%) followed by levofloxacin (56.8%), norfloxacin and gatifloxacin (each 48.6%) (Table 2). All 120 quinolone-resistant isolates were simultaneously resistant against 2 or more quinolones (Table 3).

**Table 2:** Quinolone resistance rates of all 185 *Pseudomonas aeruginosa* isolates.

Antibiotic	Susceptible n (%)	Resistant n (%)
Gatifloxacin	95 (51.4)	90 (48.6)
Norfloxacin	95 (51.4)	90 (48.6)
Ciprofloxacin	75 (40.5)	110 (59.4)
Ofloxacin	100 (54.1)	85 (45.9)
Levofloxacin	80 (43.2)	105 (56.8)

**Table 3:** Quinolone co-resistance rates of all 120 quinolone-resistant *Pseudomonas aeruginosa* isolates.

Antibiotic	Resistance rate n (%)
Ciprofloxacin-Norfloxacin-Levofloxacin	30 (16.2)
Gatifloxacin-Ciprofloxacin-Ofloxacin-Levofloxacin	30 (16.2)
Gatifloxacin-Norfloxacin-Ciprofloxacin-Ofloxacin-Levofloxacin	40 (21.6)
Gatifloxacin-Norfloxacin-Ofloxacin-Levofloxacin	5 (2.7)
Gatifloxacin-Norfloxacin-Ciprofloxacin-Ofloxacin	10 (5.4)
Norfloxacin-Gatifloxacin	5 (2.7)

### Antibiotic resistance rates of quinolone-susceptible and quinolone-resistant isolates

The *P. aeruginosa* isolates showed the most predominant resistance against third generation cephalosporins: ceftriaxone (78.4%) and ceftazidime (76.2%). Antibacterial resistance rates of quinolone-susceptible and quinolone-resistant isolates are summarized in Table 4. Aztreonam, cefepime, and tobramycin were the most effective antibiotics against quinolone-susceptible and quinolone-resistant isolates. There was no significant differences between the quinolone-susceptible and quinolone-resistant isolates in term of antibiotic resistance rates. The rates of MDR, extensively drug-resistant (XDR), and pandrug-resistant (PDR) isolates were, 78.4% (n = 145), 8.1% (n = 15), and 0.0 % (n = 0), respectively. The MDR isolates showed 10 various resistotypes (Table 5).

**Table 4:** Antibiotic resistance rates of quinolone-susceptible and quinolone-resistant *Pseudomonas aeruginosa* isolates.

Antibiotics	Quinolone-susceptible isolates n: 65 (35.1%)		Quinolone-resistant isolates n: 120 (64.9%)		Total n: 185 (100.0%)		*P*- value
	S n (%)	R n (%)	S n (%)	R n (%)	S n (%)	R n (%)	
Aztreonam	30 (46.2)	35 (53.8)	50 (41.7)	70 (58.3)	80 (43.2)	105 (56.8)	0.6413
Cefepime	25 (38.5)	40 (61.5)	36 (30.0)	84 (70.0)	61 (33.0)	124 (67.0)	0.2555
Ceftriaxone	15 (23.1)	50 (76.9)	25 (20.8)	95 (79.2)	40 (21.6)	145(78.4)	0.7129
Ceftazidime	17 (26.2)	48 (73.8)	27 (22.5)	93 (77.5)	44 (23.8)	141 (76.2)	0.5913
Tobramycin	25 (38.5)	40 (61.5)	40 (33.4)	80 (66.7)	65 (35.1)	120 (64.9)	0.5209
Piperacillin/tazobactam	22 (33.8)	43 (66.2)	27 (22.5)	93 (77.5)	49 (26.5)	136 (73.5)	0.1165
Gentamycin	22 (33.8)	43 (66.2)	25 (20.8)	95 (79.2)	47 (25.4)	138 (74.6)	0.0761
Imipenem	24 (36.9)	41 (63.1)	35 (29.2)	85 (70.8)	59 (31.9)	126 (68.1)	0.3225
Piperacillin	19 (29.2)	46 (70.8)	30 (25.0)	90 (75.0)	49 (26.5)	136 (73.5)	0.6014
Amikacin	21 (32.3)	44 (67.7)	33 (27.5)	87 (72.5)	54 (29.2)	131 (70.8)	0.5026

*S* susceptible, *R* resistant.

**Table 5:** Resistotypes of Pseudomonas aeruginosa isolates based on studied antibiotic groups.

Resistotypes (*n*, %)	Antibiotic classes														
	C1			C2	C3					C4			C5		C6
	TN	AN	GEN	IMI	CIP	LEV	OFL	NOR	GAT	CAZ	CFP	CRO	PIP	PTZ	AZT
R1 (30, 16.2)	R	R	R	R	R	R		R		R		R	R	R	R
R2 (30, 16.2)	R	R	R	R	R	R	R		R	R	R	R	R	R	R
R3 (25, 13.5)		R	R	R	R	R	R	R	R	R	R	R	R	R	
R4 (5, 2.7)	R	R		R		R	R	R	R	R	R		R		R
R5 (10, 5.4)	R	R	R	R	R		R	R	R	R	R	R	R		
R6 (5, 2.7)	R		R	R				R	R	R		R	R	R	R
R7 (15, 8.1)	R	R	R	R	R	R	R	R	R	R	R	R	R	R	R
R8 (16, 8.6)	R		R							R	R	R	R	R	R
R9 (5, 2.7)	R		R	R						R	R	R		R	
R10 (4, 2.2)	R										R	R		R	

C1, aminoglycosides; C2, carbapenems; C3, fluoroquinolones; C4, cephalosporins; C5, penicillins; C6, monobactams; C10, IMI, imipenem; CIP, ciprofloxacin; AN, amikacin; AZT, aztreonam; CFP, cefepime; CAZ, ceftazidime; CRO, ceftriaxone; GAT, gatifloxacin; NOR, norfloxacin; GEN, gentamycin; LEV, levofloxacin; OFL, ofloxacin; PIP, piperacillin; PTZ, piperacillin/tazobactam; TN, tobramycin.

### Presence of PMQR genes

Out of 120 fluoroquinolone-resistant *P. aeruginosa* strains, 46 (38.3%) isolates were positive for the *qnr* genes by PCR screening. The results of molecular assay indicated that *qnrB* gene was the most predominant 29.2% (n = 35), followed by *qnrA* 25.8% (n =31), and *qnrS* gene 20.8% (n = 25), respectively. The *qnr* genes were occurred in 37.5% (n = 45/120) of isolates, simultaneously. The *qnrA* gene was found to coexist with *qnrB* in 20 (16.7%) and coexist with *qnrS* in 11 (9.2%) isolates, respectively. Also, 10 (8.3%) isolates harbored both *qnrB* and *qnrS*, and 4 (3.3%) isolates harbored the *qnrA*, *qnrB*, and *qnrS* genes, simultaneously. The *qnrC*, *qnrD*, *qepA*, and *aac(6′)-Ib-cr* genes were not detected in studied isolates.

## Discussion

In the Iran, few studies on the incidence of quinolone-resistant *P. aeruginosa* and its plasmid-mediated mechanisms have been reported. In this regard, we designed the present study to the better understanding of molecular and epidemiological aspects of quinolone resistance pattern of clinical *P. aeruginosa* isolates in Ahvaz, southwest Iran. In this study, 185 clinical *P. aeruginosa* isolates were investigated. The most strains were isolated from wound (37.3%), urine (21.6%), and blood (19.4%). Also, the majority of them were collected from Burn, Urology, and ICU ward. In agreement with these findings, a previous report from Iran by Izadi Pour Jahromi et al.^15^ from Iran, isolated the most *P. aeruginosa* strains from wound and urine. Also, they showed the more incidence of *P. aeruginosa* in Burn, Pediatric, and ICU wards. Also, Shahraki Zahedani et al.^16^ reported the most prevalent *P. aeruginosa* isolates in urine (17.44%), wound (24.41 %), and blood (33.72%) samples.

This research revealed a total quinolone resistance rate of 64.9% among clinical *P. aeruginosa* isolates that was higher than the previous reports from Saudi Arabia (42.4%) and Egypt (57.2%)^13,17^. In this study, the resistance rates against five tested quinolones including gatifloxacin, norfloxacin, ciprofloxacin, ofloxacin, and levofloxacin varied from 45.9% to 59.4%. The antimicrobial susceptibility testing revealed that the most effective quinolone in the present study was ofloxacin, while more of our isolates were resistant to ciprofloxacin. In contrast to the current findings, Rajaei et al.^18^ from Iran reported the ciprofloxacin as the most effective antibiotic against clinical *P. aeruginosa* isolates. In another study by Adwan et al.^14^ from Palestine who investigated 11 clinical *P. aeruginosa* isolates, 100.0 % of them were resistant against norfloxacin and ciprofloxacin. El-Badawy et al.^13^ who investigated seven quinolone antibiotics including nalidixic acid, ciprofloxacin, norfloxacin, ofloxacin, levofloxacin, gemifloxacin, and moxifloxacin against *P. aeruginosa* isolates, disclosed resistance rates ranging from 28.3% to 41.3%. In their study, the gemifloxacin with 28.3% and nalidixic acid with 41.3% resistance rates were the most and the less effective quinolones, respectively that were not evaluated in the current research.

In this study, although the resistance to all tested antibiotics was more than 50.0%, aztreonam, tobramycin, cefepime, and imipenem were among the most effective drugs against both quinolone-resistant and quinolone-susceptible strains. However, our isolates showed a high resistance rates (more than 70.0%) against other aminoglycosides (gentamycin, amikacin), cephalosporins (ceftazidime, ceftriaxone), and penicillins (piperacillin, piperacillin/tazobactam). Previous studies from Iran have approved the high resistance rates of the *P. aeruginosa* to a wide range of antibiotics, which was similar to current results^19,20^. In contrast to these findings, Brzozowski et al.^21^ from Poland reported a lower resistance rates for ciprofloxacin (39.1%), amikacin (30.7%), cefepime (42.6%), ceftazidime (33.2%), gentamycin (37.6%), piperacillin/tazobactam (39.6%), tobramycin (38.1%), and imipenem (67.8%) in clinical *P. aeruginosa*. These discrepancies could be due to differences in the geographical regions and diversity of antibiotic prescription patterns, as well as the lack of a comprehensive and organized monitoring program for the proper use of antibiotics in several countries.

In recent years, the prevalence of carbapenem-resistant Gram-negative bacteria has increased worldwide. This is a unique clinical problem, as these drugs are long regarded to be the most active and powerful treatment for the infections caused by MDR bacteria. In the current study, 68.1% of *P. aeruginosa* isolates were resistant to imipenem. In line with our findings, previous reports by Farajzadeh Sheikh et al.^19^ (90.7%) and Tarafdar et al.^20^ (95.8%) from Iran, stated a high resistance rate against carbapenems in the clinical *P. aeruginosa* isolates. This may be due to the presence of metallo-β-lactamase or carbapenemase enzymes and upregulation of different efflux pumps in these strains^22^.

Another remarkable result of the current study was the high occurrence of MDR *P. aeruginosa* (78.4%) that was greater than previous indicated statistics from Iran (31.4%), Poland (48%)^28^, and Egypt (66.6%)^23^. However, the XDR rate (8.1%) was lower than a report by Shahraki Zahedani et al.^16^ from Iran (12.3%). No PDR isolate was detected in this study. In another study by Pérez et al.^24^, who investigated 53 *P. aeruginosa* isolates from Greece, Italy, and Spain, a total of 30.2% MDR, 35.8% XDR, and 3.8% PDR strains were reported. In our region, incorrect antibiotic prescriptions might be a contributing factor to this higher prevalence of MDR isolates.

This study was the first report on the presence of the *qnr* genes in quinolone-resistant clinical *P. aeruginosa* isolates in patients from southwest Iran. According to our findings, 38.3% of isolates were positive for the *qnr* genes, from which 29.2%, 25.8%, and 20.8% isolates had the *qnrB*, *qnrA*, and *qnrS* genes, respectively. So far, despite the importance of the subject, few studies have addressed this issue.

In a study by Michalska et al.^25^ from Poland, the *qnrB* with a frequency of 20.0% was the only detected gene among clinical *P. aeruginosa* isolates. In contrast to the current research, they did not find the *qnrA* and *qnrS* genes. Also, our findings significantly differed from the results reported by Molapour et al.^26^ from Iran who did not find any *qnr* gene in 149 quinolone-resistant *P. aeruginosa* that were isolated from burn patients. In another study by Cayci et al.^27^ from Turkey, *qnrA*, *qnrB*, and *qnrS* genes were not detected in *P. aeruginosa* isolates.

The *qnrB* gene was the most common *qnr* type in our region. However, a previous reports by Rajaei et al.^18^ from Kerman city, Iran indicated the *qnr*A gene as the predominant PMQR (16.6%). Also, in comparison with our findings, they showed a lower prevalence rates for *qnrB* (13.3%) and *qnrS* (11.6%). Moreover, Saleh et al.^17^ from Egypt, showed a lower prevalence rates for *qnr* genes in comparison with our findings. In their study, the total prevalence rate for *qnr* genes was equal to 4.5%. They detected the *qnrB* and *qnrS* in 1.8% and 2.7% of *P. aeruginosa*, respectively. El-Badawy et al.^13^ from Saudi Arabia reported a much higher occurrence rate for *qnrS* (79.5%) than our study. Also, in the previous studies from Egypt, Iraq, and China, the *qnrS* has been recorded as the major quinolone resistant gene among MDR *P. aeruginosa* strains^17,28,29^.

In this study, the *qnrC*, *qnrD*, *qepA*, and *aac(6′)-Ib-cr* genes were not detected in studied isolates. In contrast to our report, El-Badawy et al.^13^ and Jiang et al.^29^ reported a prevalence of 79.5% and 0.9% for *qnrD*, respectively. However, previous studies from Saudi Arabia^13^, Egypt^17^, and China^29^ were not detected the *qepA* gene that was in line with the current study. The *aac(6′)-Ib-cr* gene was first identified in 2003, and it has subsequently been found in a variety of regions and sources^30^. This gene has been reported in *P. aeruginosa* strains in previous studies from Saudi Arabia (71.8%)^13^, Iran (8.3%)^18^, and China (1.9%)^29^. Also, in a report by Molapour et al.^26^ from Iran, all 149 *P. aeruginosa* isolates harbored *aac(6*′*)-Ib-cr* gene that was inconsistent with our result.

In the current study, the *qnr* genes were occurred in 37.5% (n = 45/120) of isolates, simultaneously. In line with these results, the coexistence of PMQR genes has been reported previously in *P. aeruginosa* isolates from various countries including Saudi Arabia and China^13,29^.

We found that 74 (61.7%) fluoroquinolone-resistant clinical isolates were negative for the *qnrA*, *qnrB*, , *qnrC*, *qnrD*, *qnrS*, *qepA*, and *aac(6′)-Ib-cr* genes. The quinolone resistance phenomenon in these isolates may be due to the other *qnr* genes like *qnrE* and *qnrVC*, or recently introduced *crpP* gene that encodes a ciprofloxacin modifying enzyme CrpP that were not investigated here^12,31^. After being discovered in a Brazilian *Vibrio cholerae* strain in 1998, *qnrVC* gene is now more often linked with bacteria that live in the aquatic environment^31,32^. This gene has different alleles. In two recent studies by Khan et al.^31^ and Lin et al.^33^ the prevalence rates of 12.0% and 2.3% were reported for this gene in clinical *P. aeruginosa*. Also, Khan et al.^31^ reported a prevalence of 63.0% for *crpP* gene in corneal *P. aeruginosa* isolates. So far, these genes have not been investigated and reported in any bacteria from Iran.

As the majority of quinolone-resistant *P. aeruginosa* strains in this study lack the PMQR genes, it is recommended to investigate the other aforesaid mechanisms to shed light on the precise molecular epidemiology of these isolates.

In conclusion, considering that the antibiotic resistance profiles constantly differ in each area and hospital setting, the periodic surveillance program is very crucial for each country to determine the most appropriate treatment choices. Based on our results, the ofloxacin was the best quinolone for the treatment of *P. aeruginosa* infections. Also, aztreonam, cefepime, and tobramycin could be suitable alternative treatment when there are restrictions or inhibitions on the use of quinolones. Also, the high frequency of MDR *P. aeruginosa* justifies the need to develop a monitoring program to reduce this occurrence and control the more spread of these strains in our region. This research was the first work in southwest Iran which adds to our knowledge of how *P. aeruginosa* withstand quinolones. The *qnrB* was the most PMQR determinant in clinical *P. aeruginosa* isolates from southwest Iran, while the *qnrC*, *qnrD*, *qepA*, and *aac(6′)-Ib-cr* genes were not detected. Other possible mechanisms of resistance should also be studied for better characterization of quinolone-resistant *P. aeruginosa* isolates. Lack of evaluation of chromosomal mutations in the QRDR region and failure to use whole-genome sequencing to provide more in-depth data on high-risk clones and other resistance genes (such as extended spectrum beta-lactamases and carbapenemases) often associated with PMQR, were some limitations of this study.

## Materials and methods

### Ethics statement

This study was approved by the Ethics Committee of the Ahvaz Jundishapur University of Medical Sciences, Ahvaz, Iran (No: IR.AJUMS.REC.1397.206). All methods were performed in accordance with the relevant guidelines and regulations of the Ethics Committee of the Ahvaz Jundishapur University of Medical Sciences, Ahvaz, Iran. The informed consent was obtained from all patients.

### Samples and patients

In this cross-sectional study during March 2017–April 2019, a total of 185 non-repetitive clinical isolates of *P. aeruginosa* were obtained from patients who referred to the four university teaching hospitals in Ahvaz, southwest of Iran. The *P. aeruginosa* isolates were sent to the laboratory of Department of Microbiology, Faculty of Medicine, Ahvaz Jundishapur University of Medical Sciences, for further confirmation and molecular assays.

### Bacterial isolation and identification

All studied samples were inoculated on 5% sheep blood agar and MacConkey agar plates and incubated aerobically at 37 °C for 24 h. The *P. aeruginosa* isolates were identified by standard bacteriological analyses such as Gram staining, catalase, oxidase, biochemical reaction on sulfur-indole-motility (SIM) agar, triple sugar iron (TSI) agar, lysine iron agar (LIA), oxidative fermentative (OF) test, growth at 42 °C, and growth on cetrimide agar^34^. All culture media were purchased from Merck Co., Darmstadt, Germany. The suspected *P. aeruginosa* isolates were confirmed by polymerase chain reaction (PCR) using specific primers for *ecfX* gene as described by Sands et al. (Table 6)^35^.

**Table 6:** The characteristics of primer sequences for the detection of PMQR genes in this study.

Target gene	Sequence (5′–3′)	Product Size (bp)	Annealing (°C)	References
*ecfX*	ATGGATGAGCGCTTCCGTG TCATCCTTCGCCTCCCTG	528	58	^35^
*aac(6*′*)-Ib*	TTGCGATGCTCTATGAGTGGCTA CTCGAATGCCTGGCGTGTTT	482	56	^39^
*qnrA*	ATTTCTCACGCCAGGATTTG GATCGGCAAAGGTTAGGTCA	516	53	^39^
*qnrB*	GATCGTGAAAGCCAGAAAGG ATGAGCAACGATGCCTGGTA	476	55	^39^
*qnrC*	GGGTTGTACATTTATTGAATCG CACCTACCCATTTATTTTCA	307	50	^39^
*qnrD*	CGAGATCAATTTACGGGGAATA AACAAGCTGAAGCGCCTG	582	53	^40^
*qnrS*	GCAAGTTCATTGAACAGGGT TCTAAACCGTCGAGTTCGGCG	428	56	^39^
*qepA*	AACTGCTTGAGCCCGTAGAT GTCTACGCCATGGACCTCAC	596	55	^39^

### Antimicrobial susceptibility testing (AST)

The resistance pattern of isolates against 15 antibiotics including 5 fluoroquinolones was determined by disc diffusion method on Mueller-Hinton agar (Merck, Germany) as described by the Clinical Laboratory Standards Institute (CLSI 2017) guidelines^36^. The antibiotic disks used were as follows: amikacin (30 μg), aztreonam (30 μg), cefepime (30 μg), ceftazidime (30 μg), ceftriaxone (30 μg), ciprofloxacin (5 μg), gatifloxacin (5 μg), norfloxacin (5 μg), gentamycin (10 μg), imipenem (10 μg), levofloxacin (5 μg), ofloxacin (5 μg), piperacillin (100 μg), piperacillin/tazobactam (100 μg/10 μg), and tobramycin (10 μg) (MAST Co., Berkshire, UK). Drug-resistant patterns were defined as follows: MDR isolates (resistant to at least three antibiotics belonging to different chemical classes), XDR strains (resistant to at least one agent in all but two or fewer antimicrobial groups), and PDR strains (resistant to all antimicrobial classes)^37^. *E. coli* ATCC 25922 and *P. aeruginosa* ATCC 27853 were used as quality control strains.

### DNA extraction

DNA was extracted using boiling method as previously described^38^. Briefly, few colonies were picked up from an overnight growth (24 h) on a Mueller-Hinton agar and suspended into 500 μl of distilled water. The mixture was vortexed for 20 s and then boiled for 10 min. In the end, all samples were centrifuged at 14,000 rpm for 10 min and the supernatants were stored at − 20 °C as DNA template for polymerase chain reaction (PCR) assay. The quality and quantity of DNA (ng/µl) were evaluated by measuring the absorbance of A260 and A280 nm with a NanoDrop spectrophotometer (Thermofisher Scientific, USA). The DNA samples that had a concentration of at least 50 ng/µl, were selected for PCR.

### PCR assay for PMQR genes

The previously published PCR primer pairs were used for the detection of *qnrA*, *qnrB*, , *qnrC*, *qnrD*, *qnrS*, *qepA*, and *aac(6′)-Ib-cr* genes and the specificity of each primer was proved using the NCBI Primer-BLAST (https://www.ncbi.nlm.nih.gov/tools/primer-blast/)^39,40^. The sequences of all used primers are presented in Table 6. The PCR assay was performed under the following conditions: initial denaturation step for 4 min at 94 °C, 35 cycles of denaturation at 94 °C for 45 s, annealing for 40 s (temperature was depending on the sequence of primers), extension at 72 °C for 50 s, and a final extension step of 72 °C for 5 min. The final products were detected by electrophoresis on 1% agarose gel (70 V, 1 h) containing ethidium bromide (0.5 μg/ml) in the Tris-EDTA buffer, and the gel was seen under ultraviolet illuminator (Proteinsimple, San Jose, CA, USA). The size of PCR amplicons was estimated by the migration pattern of a 100-bp DNA ladder (Sinaclon, Tehran, Iran). For confirmation, some of PCR products were sent to Bioneer Corporation (Korea) for gene sequencing (ABI PRISM 7700 Sequence Detection System, Applied Biosystems, Foster City, CA). These products were used as control positive. Also, in each PCR run, the distilled water was used as negative control.

### Statistical analysis

Statistical data analysis was performed by the use of SPSS software version 22.0 (IBM Corporation, Armonk, NY, USA). The results were described as descriptive statistics in terms of relative frequency. The Fisher’s test was used, with a *P*-value < 0.05 deemed statistically significant.

## Data Availability

The datasets used and/or analyzed during the current study are available from the corresponding author on reasonable request.
