# Publisher Correction: Occurrence of plasmid‑mediated quinolone resistance genes in *Pseudomonas aeruginosa* strains isolated from clinical specimens in southwest Iran: a multicentral study

**DOI:** 10.1038/s41598-022-07352-8

**Published:** 2022-03-03

**Authors:** Morteza Saki, Ahmad Farajzadeh Sheikh, Sakineh Seyed‑Mohammadi, Aram Asareh Zadegan Dezfuli, Mojtaba Shahin, Maryam Tabasi, Hojat Veisi, Raziyeh Keshavarzi, Parisa Khani

**Affiliations:** 1 Infectious and Tropical Diseases Research Center, Health Research Institute, Ahvaz Jundishapur University of Medical Sciences, Ahvaz, Iran; 2 Department of Microbiology, Faculty of Medicine, Ahvaz Jundishapur University of Medical Sciences, Ahvaz, Iran; 3 Student Research Committee, Ahvaz Jundishapur University of Medical Sciences, Ahvaz, Iran; 4 Department of Medical Laboratory Sciences, Faculty of Medical Sciences, Arak Branch, Islamic Azad University, Arak, Iran; 5 Department of Virology, Faculty of Medicine, Ahvaz Jundishapur University of Medical Sciences, Arak, Iran

Correction to: *Scientific Reports* https://doi.org/10.1038/s41598-022-06128-4, published online 10 February 2022

The original version of this Article contained errors.

Affiliation 3 for Aram Asareh Zadegan Dezfuli was incorrectly labelled as a present address. The correct affiliations are listed below.

Infectious and Tropical Diseases Research Center, Health Research Institute, Ahvaz Jundishapur University of Medical Sciences, Ahvaz, Iran

Department of Microbiology, Faculty of Medicine, Ahvaz Jundishapur University of Medical Sciences, Ahvaz, Iran

Student Research Committee, Ahvaz Jundishapur University of Medical Sciences, Ahvaz, Iran

Affiliation 5 was incorrectly given as ‘5Department of Virology, Faculty of Medicine, Ahvaz Jundishapur University of Medical Sciences, Arak, Iran’. The correct affiliation is listed below.

Department of Virology, Faculty of Medicine, Ahvaz Jundishapur University of Medical Sciences, Ahvaz, Iran

Additionally, in the Results section, under the subheading ‘Bacterial isolates’,

“Based on standard bacteriological tests, overall, 185 (22.5%) clinical strains of *P. aeruginosa* were collected from four main hospitals in Ahvaz, southwest of Iran during a nine-month period.”

now reads:

“Based on standard bacteriological tests, overall, 185 (22.5%) clinical strains of *P. aeruginosa* were collected from four main hospitals in Ahvaz, southwest of Iran during a two-year period.”

In the Discussion section,

“In this study, although the resistance to all tested antibiotics was more than 50.0%, azetonium, tobramycin, cefepime, and imipenem were among the most effective drugs against both quinolone-resistant and quinolone-susceptible strains.”

now reads:

“In this study, although the resistance to all tested antibiotics was more than 50.0%, aztreonam, tobramycin, cefepime, and imipenem were among the most effective drugs against both quinolone-resistant and quinolone-susceptible strains.”

“Another remarkable result of the current study was the high occurrence of MDR *P. aeruginosa* (78.4%) that was greater than previous indicated statistics from Iran (31.4%), Poland (48%) (28), and Egypt (66.6%)^23^.”

now reads:

“Another remarkable result of the current study was the high occurrence of MDR *P. aeruginosa* (78.4%) that was greater than previous indicated statistics from Iran (31.4%), Poland (48%)^28^, and Egypt (66.6%)^23^.”

Lastly, the grant number for ‘Ahvaz Jundishapur University of Medical Sciences’ was incorrectly given in the metadata.

“OG-93118”

now reads:

“OG-9717”

The original Article has been corrected.

